# Comprehensive comparison of patient-derived xenograft models in Hepatocellular Carcinoma and metastatic Liver Cancer

**DOI:** 10.7150/ijms.46686

**Published:** 2020-10-22

**Authors:** Wei Xu, Zheng-Yun Zhao, Qi-Ming An, Bin Dong, Ang Lv, Cheng-peng Li, Xiao-Ya Guan, Xiu-Yun Tian, Jian-Hui Wu, Chun-Yi Hao

**Affiliations:** 1 Key Laboratory of Carcinogenesis and Translational Research (Ministry of Education/Beijing), Department of Hepato-Pancreato-Biliary Surgery, Peking University Cancer Hospital & Institute, Beijing, China.; 2 Department of Chemistry, Durham University, Stockton Road, Durham DH1 3LE, U.K.; 3 Department of Gastrointestinal Surgery, the Affiliated Hospital of Inner Mongolia Medical University, Hohhot 010050, China.; 4 Key laboratory of Carcinogenesis and Translational Research (Ministry of Education/Beijing), Center laboratory, Peking University Cancer Hospital & Institute, Beijing, China

**Keywords:** PDX, hepatocellular carcinoma, metastatic liver cancer, transplantation rate

## Abstract

Patient-derived xenograft (PDX) models are effective preclinical cancer models that reproduce the tumor microenvironment of the human body. The methods have been widely used for drug screening, biomarker development, co-clinical trials, and personalized medicine. However, the low success rate and the long tumorigenesis period have largely limited their usage. In the present studies, we compared the PDX establishment between hepatocellular cancer (HCC) and metastatic liver cancer (MLC), and identified the key factors affecting the transplantation rate of PDXs. Surgically resected tumor specimens obtained from patients were subcutaneously inoculated into immunodeficient mice to construct PDX models. The overall transplantation rate was 38.5% (20/52), with the HCC group (28.1%, 9/32) being lower than MLC group (56.2%, 9/16). In addition, HCC group took significantly longer latency period than MLC group to construct PDX models. Hematoxylin and eosin staining results showed that the histopathology of all generations in PDX models was similar to the original tumor in all three types of cancer. The transplantation rate of PDX models in HCC patients was significantly associated with blood type (*P*=0.001), TNM stage (*P*=0.023), lymph node metastasis (*P*=0.042) and peripheral blood CA19-9 level (*P*=0.049), while the transplantation rate of PDX models in MLC patients was significantly associated with tumor size (*P*=0.034). This study demonstrates that PDX models can effectively reproduce the histological patterns of human tumors. The transplantation rate depends on the type of original tumor. Furthermore, it shows that the invasiveness of the original liver cancer affects the possibility of its growth in immunodeficient mice.

## Introduction

Liver cancer can be divided into two major categories: primary liver cancer (PLC), which arises from the liver, and secondary liver cancer, also known as metastatic liver cancer (MLC), which is formed initially from other parts of the body and then spreads to the liver. Hepatocellular carcinoma (HCC), the major type of PLC, is the fourth most common cause of cancer-related death worldwide 1. Liver cancer management includes surgery, chemotherapy, ablation therapy and targeted medicine treatment. Although surgery is the most effective treatment, a large number of patients missed the surgery opportunity when diagnosed. Many of these patients with unresectable HCC or MLC have to take the multi-kinase inhibitor Sorafenib as alternative treatment for survival improvements, which has adverse effects that might be harmful for patients' wellbeing 2, 3. The quest for an optimized, well-tolerated, minimally invasive, cost-effective therapy for liver cancer management remains an unmet clinical need and active research topic. Therefore, relevant preclinical animal models are crucial for the development of innovative approaches for liver cancer therapy, such as gene therapy and immunotherapy.

Patient-derived xenograft (PDX) models have been commonly used for developing new drugs and guiding individualized medicine 4. PDX models are established by subcutaneously or orthotopically transplanting tumor tissues of patients into immunodeficient mice such as NOD/SCID (non-obese diabetic/severe combined immunodeficiency) mice, which can reproduce the tumor microenvironment similar to primary tumors in human bodies 5-8. PDXs of various tumors have been constructed, such as colorectal cancer 9, breast cancer 10, and renal cell carcinoma 11 over the last few years. In our previous study, we have revealed the PDXs of pancreatic cancer could well recapitulated the histologic, expression and biological characteristics of the corresponding primary tumors 12. However, the low success rate, long tumorigenesis period and high cost are huge obstacles limiting the widespread of PDXs in clinical treatment. Therefore, how to solve these problems and make transplant easier to operate remain our task in the long research path. In this study, we focus on the difference of patient-derived xenograft models in hepatocellular carcinoma and metastatic liver cancer, in hope that the findings can provide useful message for improving the success rate of PDX.

## Material and Methods

### Patients and samples

Surgically resected tumor specimens (n=52) and paired adjacent normal tissues were obtained from patients diagnosed with HCC, MLC or CRC, who underwent surgery at the Peking University Cancer Hospital from November 2013 to January 2014. Each specimen measured approximately 1×1×1 cm in dimension. Fresh tumor specimens were divided into three parts: one part was transferred to antibiotic-containing Dulbecco's modified Eagle's medium (DMEM; Gibco BRL, Life Technologies, Grand Island, NY, USA) for tumor transplantation; one part was fixed in 4% formalin for Hematoxylin-eosin (H&E) staining; and the rest was immediately transferred to liquid nitrogen and stored at -80°C for future studies. Each part measured approximately 5×5×5 mm. This study was approved by the medical ethics committee of Peking University Cancer Hospital and was carried out in accordance with the approved guidelines.

### Establishment of PDX models

All tumor specimens from the patients (termed F0) were subcutaneously inoculated into the right buttock of 5-week-old NOD/SCID mice (Beijing HFK Bio-Technology Co., LTD, Beijing, China), weighting 18-20 g. Tumor specimens were cut into small pieces (less than 1 mm^3^) and mixed with Matrigel (100 µl per sample) (BD Biosciences, Heidelberg, Germany) for 20 seconds at room temperature, immediately prior to xenotransplantation. Tumor growth was measured twice a week by a Vernier caliper, using the following formula 13: tumor volume = (length×width^2^)/2.

When the tumor size reached approximately 1000 mm^3^, the xenograft mouse was sacrificed under anesthesia, and the tumor was excised and divided into three parts, identical to the procedure followed for the human specimens. The DMEM-stored portion was used to re-inoculate the mice to obtain subsequent generations containing the tumor mass. This generation of mice, receiving the patient tumor transplant, was termed F1. Similarly, the following generations were termed F2, F3…Fn respectively 14. Mice were kept in the animal facilities of the Peking University Cancer Hospital and maintained in specified pathogen-free conditions. Animals were exposed to 12 h light/12 h darkness cycles and provided with standard food and water ad libitum. All procedures were performed under sterile conditions and carried out in accordance with the Guide for the Care and Use of Laboratory Animals of the National Institutes of Health.

### Statistical analysis

The sample size of this study was determined using R [R Core Team (2019), Version 3.6.1] with RStudio (Version 1.2.5019) and Pwr Package prior to commencing the experiment with a view of obtaining a power of 80%. Calculation was based on the lines of Cohen (1988) using in particular the same notations for effect sizes 15. The estimated sample size needed to reach 80% power on the 0.05 significance level (two-sided test) with a correlation coefficient (*r*) smaller than 0.5, leaded to a sample size of 28 study subjects.

Unpaired 2-tailed t tests were used for group comparisons after verifying normality and homogeneity of variance. The relationship between clinicopathological characteristics and transplantation rate of xenografts was analyzed using the chi-square test (all patients when more than 40 samples were enrolled) and Fisher's exact test (HCC and MLC patients when less than 40 samples were enrolled). All statistical analyses were performed using IBM SPSS 25.0 software (SPSS Inc., Chicago, IL, USA).

In addition, all patients were evaluated for overall survival (OS) and progression-free survival (PFS). OS was defined as the elapsed time between the inclusion date and death due to any cause. PFS was defined as the elapsed time between the date of inclusion and the date of tumor progression. During the study, all patients did not die within 30 days of surgery. GraphPad Prism (version 7.0) was used to produce Kaplan-Meier survival curves and log-rank test was used to estimate the differences in both OS and PFS analyses. *P* values less than 0.05 (*p*<0.05) were considered to be statistically significant.

## Results

### Comparison of PDXs transplantation rates and latency period among different types of xenografted tumors

During the course of the study, a total of 80 generations of PDXs have been produced with 52 individual patient tumors, 20 (38.5%) of which were successfully generated and obtained as first generation of PDXs (F1). Transplantation rate of HCC (28.1%) was the lowest compared with CRC (50.0%) and MLC (56.2%) (Table 1). Compared with MLC group, HCC group displayed a significantly longer latency period (Table 1). In addition, successful engraftment of F2 PDXs took significant less time than F1 PDXs in MLC group, which was consistent with our previous finding in pancreatic cancer study 12. However, the latency period results did not show significant differences between F1 and F2 PDXs in the whole group.

### Comparison of histopathological characteristics

**Figure 1:**
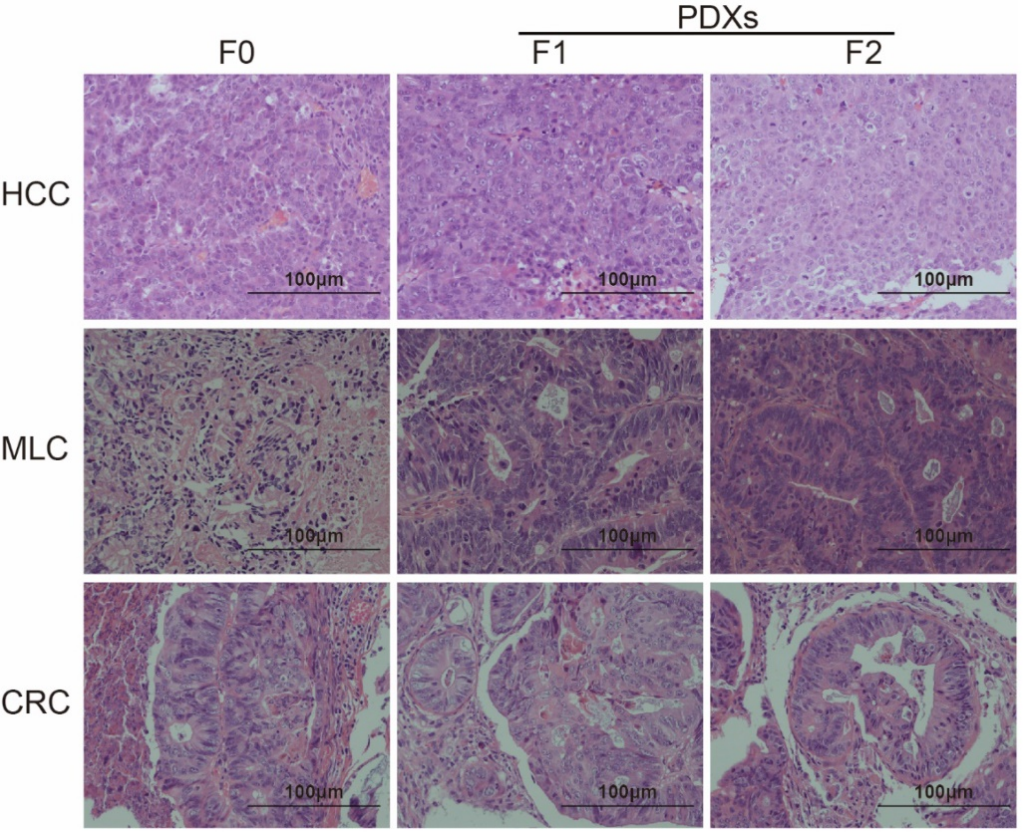
PDX generation in original tumors and NOD/SCID mice. Representative H&E staining results of tumors from HCC, MLC and CRC patients and their two corresponding passages of xenografts (×200). Pathological features of tumor could be conserved within two generations of passage in immunodeficient mice (F0 represents tumor from patient, F1 and F2 represent tumor from PDX line).

H&E staining was performed to compare the histopathology between patient tumor and its mouse avatar models. Differentiation was judged by two independent pathologists. The results showed that both F1 and F2 PDXs could preserve tumor pathological pattern of different tumor types (Figure 1).

### Correlation between transplantation rate of F1 PDXs and clinicopathological characteristics

Our results showed that the transplantation rate of PDXs was statistically significant associated with blood type (*P*=0.008), TNM stage (*P*=0.008), peripheral blood CEA (*P*=0.015) and CA19-9 level (*P*=0.017) when total patients were enrolled in the statistical analysis (Table 2).

**Figure 2:**
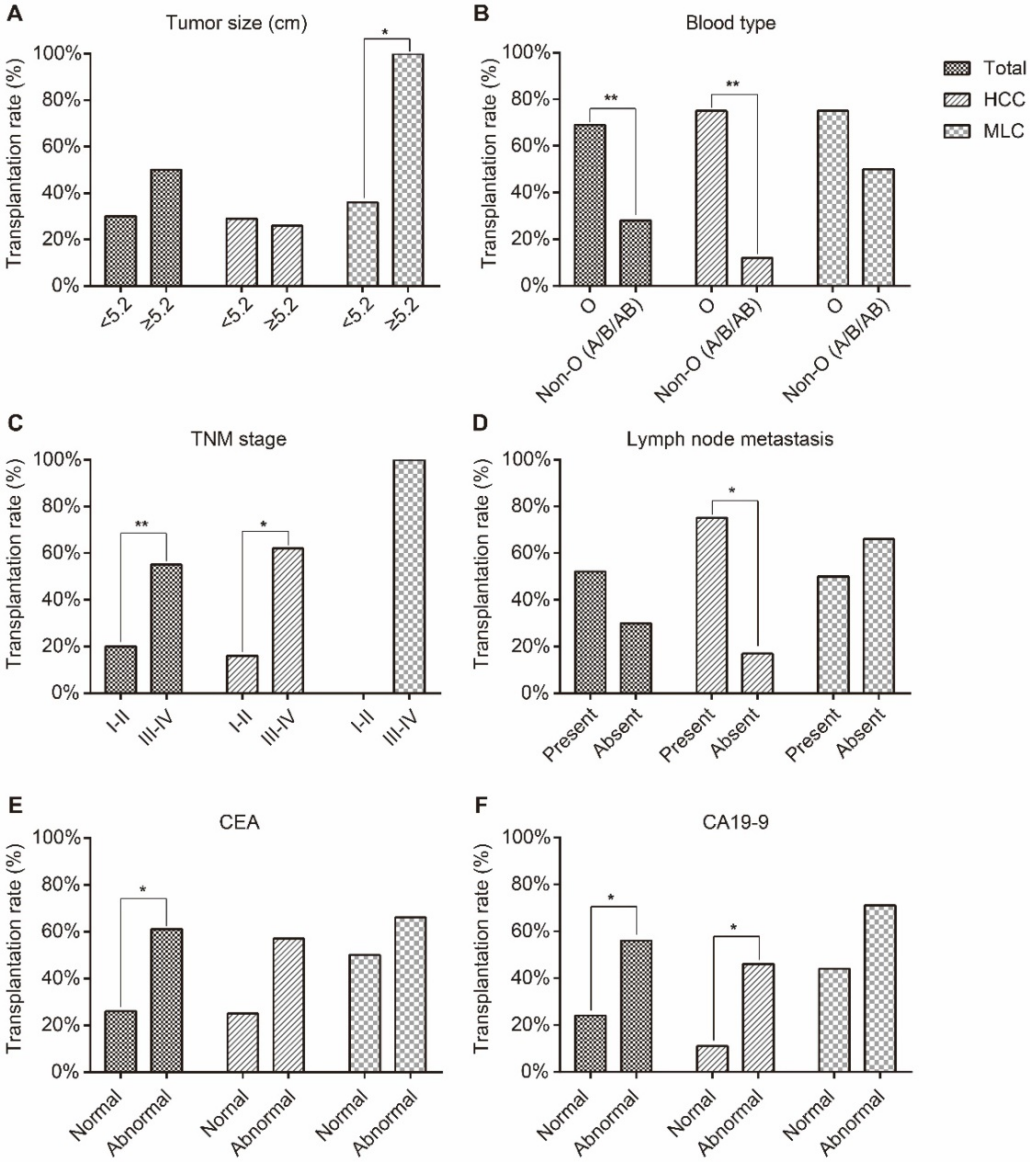
The correlations between transplantation rate and clinicopathological parameters. (**A**) Tumor size, (**B**) blood type, (**C**) TNM stage, (**D**) lymph node metastasis, (**E**) CEA and (**F**) CA19-9 level. The transplantation rate of HCC xenografts was statistically significant associated with blood type, TNM stage, lymph node metastasis and peripheral blood CA19-9 level. The transplantation rate of MLC xenografts was statistically with tumor size. * represents *p*<0.05, ** represents *p*<0.01.

Results of HCC patients showed that the transplantation rate of PDXs was statistically significant associated with blood type (*P*=0.001), TNM stage (*P*=0.023), lymph node metastasis (*P*=0.042) and peripheral blood CA19-9 level (*P*=0.049) (Table S1 and Figure 2).

Results of MLC patients showed that the transplantation rate of PDXs was statistically significant associated with tumor size (*P*=0.034) (Table S2 and Figure 2).

Furthermore, we analyzed the correlations between transplantation rate of PDXs and HBV-related antigen/antibody of HCC patients. The results showed that there were no statistically significant correlations between transplantation rate of F1 PDXs and any of the HBV-related antigens or antibodies (Table S3), which were consistent with the result of history of hepatitis virus infection (Table S1).

### Survival analysis

**Figure 3:**
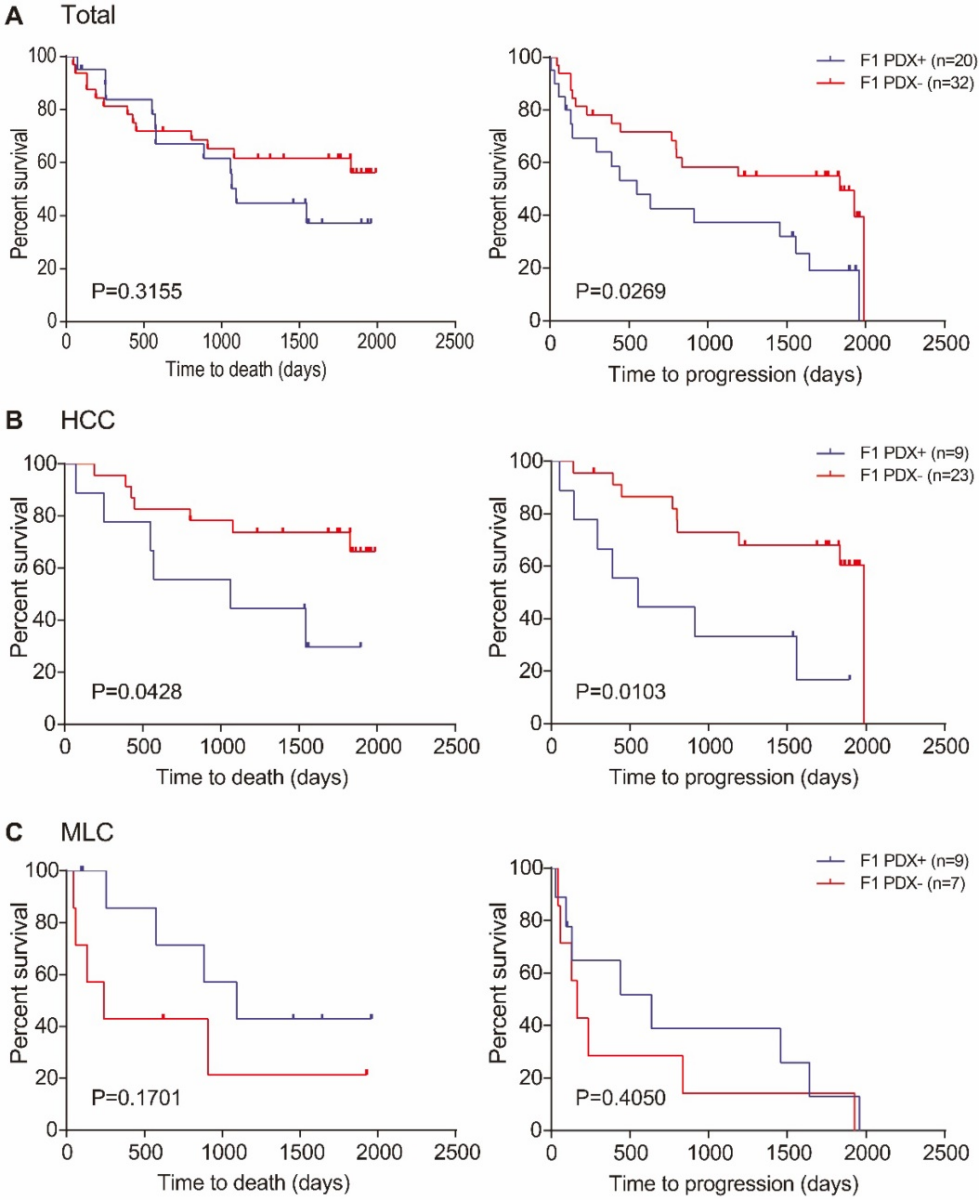
OS and PFS in relation to transplantation status of patients. (**A**) OS (left) and PFS (right) of all patients with and without success engraftment; (**B**) OS (left) and PFS (right) of HCC patients with and without success engraftment; (**C**) OS (left) and PFS (right) of MLC patients with and without success engraftment. Log-rank *p*-values are shown in each of the result.

During the diligent follow-up of more than five years, the median follow-up time was 551 days (range: 44-1829 days) for deceased patients and 1795 days (range: 96-1988 days) for patients still alive after follow-up study. The median OS in all of the 52 patients was 1829 days (range: 44-1988 days). OS results showed that there was no significant difference between the median OS in patients with successful F1 PDX transplantation and patients with failed F1 PDXs (*P*=0.3155), Figure 3A. Similar result was found in MLC patients, (*P*=0.1701) Figure 3C. However, the median OS in patients with failed F1 PDXs transplantation was significantly superior to those with successful F1 PDX transplantation, (*P*=0.0428) Figure 3B.

PFS results showed significant differences between patients with successful and failed F1 PDX transplantation, both in total and HCC patients (*P*=0.0269, *P*=0.0103, respectively) Figure 3A and B, respectively. However, no detectable difference was found in MLC patients, (*P*=0.4050) Figure 3C.

## Discussion

To thoroughly understand liver cancer and its translation into effective treatment, it is essential to establish an appropriate human preclinical model to capture the heterogeneity of cancer. Most of the tumors in liver cancer animal models are from drug-induced liver cancer or human cancer cell line derived animal models currently. However, the main drawback of these animal models is lack of both phenotypic and genetic heterogeneity found in the original tumors 16. Importantly, cancers consist of a continuously evolving heterogeneous cell mass and not all cells within a tumor contribute equally to their progression 17. Therefore, animal models possessing the characteristic of patient tumors that could accurately mimic human cancers are urgently needed in cancer research. Surgically derived primary clinical tumor samples can be grafted into immunodeficiency mice to construct PDX models. In such models, tumor architecture and the relative proportion of cancer cells and stromal cells are maintained to a large extent, which yield better resemblance to the original tumors 16, 18. In our study, the successful engraftment rate of F1 PDXs of all samples was 38.5%, which is slightly lower than the reported previously around 40%-60% 19-22, which was attributed to the lowest successful engraftment rate of HCC samples (28.1%). In addition, it took longer latency period of time to establish F1 PDXs of HCC than other tumor types. Furthermore, successful engraftment rate of F2 PDXs of HCC was also lower than CRC and MLC. These results revealed that the engraftment rate of PDXs was different according to implanted tumor types. PDX models are currently established in variety of cancers, including colorectal 23, pancreatic 24, breast 25, lung 26, prostate 27 and ovarian cancer 28. However, reports about PDX models of HCC were rare. Recently a study reported a 20% successful engraftment rate in a cohort sample of 14 HCC patients 29, another study with a larger sample size reported that only 11 successfully engrafted from 54 human HCC needle biopsies 30. The overall success rate of HCC in our study was slightly higher than researchers reported before.

Comparison of H&E staining results showed that PDXs could reproduce the histological morphology and pathology of all types of tumor (Figure 1). In our previous study, we found PDX lines could preserve the histological characters of pancreatic cancer from patients within at least three generations 12. Only two generations were established in this study, while all F2 PDXs showed well recapitulate the histological characters of their original tumors. Therefore, we assume that PDX models could resemble the histological characters of various primary tumors during passage in PDX lines.

Back to 1953, a hall mark study by Helene Toolan demonstrated that tumor xenografts were possible to establish in immune repressed hosts 31. After nearly 20 years, researchers found that anti-lymphocyte serum could improve the percentage of viable patient-derived tumor grafts slightly 32. Following these studies, researchers managed to construct severely immunodeficient mice such as the non-obese diabetic/severe combined immunodeficiency (NOD/SCID) mice, which homozygous for the severe combined immune deficiency spontaneous mutation (*Prkdc^scid^*, commonly referred to as *scid*) with NOD/ShiLtSz background. NOD/SCID mice are characterized by an absence of functional B and T cells, lymphopenia, hypogammaglobulinemia, and a normal hematopoietic microenvironment, normal antigen-presenting cell, myeloid, and natural killer (NK) cell functions are also extremely low 33, 34. “Leak” or *scid* leakiness - mice that have serum Ig levels greater than 1 μg/ml - is highly strain dependent, increases with age, and is higher in mice housed under non-SPF (specified pathogen free) conditions 35, 36. NOD/ShiLtSz strain has the lowest *scid* leakiness rate among several common genetic backgrounds such as C57BL/6J, BALB/cBy, C3H/HeJ and NOD/ShiLtSz 37. In this study, young mice (weighting 18-20g) were selected and raised in an SPF condition under strict sterile procedure to avoid leakiness to the great extent.

Absence of immune system in these mice allows for higher engraftment rates. Although successful engraftment rate has been remarkably improved by applying severely immune deficient mice, there are still a great number of the patient tumors that couldn't grow in mice 22, 29, 38. To provide useful evidence for improving engraftment rates, the influencing factors of engraftment rate were carefully investigated in our study. In this study, transplantation rate was significantly related to blood type, TNM stage, CEA and CA19-9 level in the whole cohort of patients (Figure 2, Table 2). TNM stage, CEA and CA19-9 are associated with malignant degree of tumor 39, 40. These results showed that the invasiveness of the original tumor affects the possibility of its growth in immunodeficient mice. The fact that transplantation rate of MLC was higher than HCC may also confirmed this conclusion. In the cohort of HCC patients, the factors associated with aggressive degree such as TNM stage, lymph node metastasis and CA19-9 level, were similar with the results found in whole cohort of patients. Furthermore, failed-PDX donors of HCC patients had a favorable prognosis compared with successful-PDX donors. The survival analysis results further validate the fact that the invasiveness of the original tumor affects the possibility of its growth in immunodeficient mice. Although PDX models are frequently used for cancer research, few studies have reported relevant clinical factors that may affect transplantation rate. Therefore, it is very important to reveal clinicopathological parameters that may relate to PDX transplantation rate.

To our surprise, patients with blood type O were more easily to grow their tumors in mice avatars (whole, *p*=0.008, Table 2; HCC, *p*=0.001, Table S1; MLC, *p*=0.585, Table S2; Figure 2). The ABO blood group has been found to be associated with the risk of multiple malignancies, including gastrointestinal tract cancers 41 and liver cancer^42^. The ABO gene is located on chromosome 9q34 encoding several glycosyl transferases that add sugar residues to the H(O) antigen to form ABO antigens. An association between polymorphisms at the ABO gene locus and circulating levels of several important adhesion molecules such as soluble intercellular adhesion molecule (ICAM)-1 and tumor necrosis factor-alpha was reported previously 43, 44. Researchers found that the expression of soluble ICAM-1 was significantly reduced in patients with non-O blood group compared to the expression in those with blood group O. The decreased soluble ICAM levels in patients may promote metastatic spread of tumors, therefore related with a poor prognosis 45. Although those researches demonstrated there may be differences among ABO blood groups, the exact underlying mechanism by which ABO blood group influence tumorigenesis and development remains unclear. Additional experimental studies are needed to unravel the pathogenic mechanisms linking ABO blood types with transplantation rate.

Although PDX models recapitulate tumor tissue more closely than cancer cell lines, they are usually generated from a small amount of primary tumor. The limitation of grafts could not capture the full heterogeneity of the original tumor 46. Another limitation of this study is that the immunohistochemical (IHC) study was not applied. We observed not more than one kind of cancer, the primary tumors of MLC also consisted with several tumor types. The pathological features of various types of tumors are different from each other. Therefore, only comparison of pathological pattern through H&E staining method was designed prior to our experiments. We will check the expression patterns of key factors by multiple detection methods such as IHC, real-time PCR and high-throughput sequencing in the following study. Furthermore, sample size of our study was relatively small and only two generations of PDXs were studied in this research, we will construct more mice avatar models and studied more generations of PDX lines in the future to get more in-depth data. Nevertheless, our research provides a comprehensive and objective basis for the study of PDX.

## Supplementary Material

Supplementary tables.

## Figures and Tables

**Table 1:** Primary and secondary engraftment outcomes for all tumor types

Tumor histologic type	All tumor types (n=52)	CRC (n=4)	HCC(n=32)	MLC (n=16)
**F1 engraftment results**				
No growth	32 (61.5%)	2 (50%)	23 (71.9%)	7 (43.8%)
Success	20 (38.5%)	2 (50%)	9 (28.1%)	9 (56.2%)
Latency period, days	67.7±34.1	45±12.7	88.2±34.9^*^	52.2±25.4
**F2 engraftment results**				
No growth	7 (43.8%)	0 (0%)	5 (83.3%)	1 (12.5%)
Success	9 (56.2%)	2 (100%)	1 (16.7%)	7 (87.5%)
Latency period, days	45.0±19.6	66.5±36.1	34±0.0	38.9±9.8^#^

CRC, colorectal carcinoma; HCC, hepatocellular carcinoma; MLC, metastatic liver cancer; *p<0.05 compared with MLC group. ^#^*p*<0.05 compared with F1 PDXs group.

**Table 2:** Correlations between transplantation rate and clinicopathological parameters of all patients

Clinicopathological features	No. of patients (%)	Successful engraftment (%)	*P*
**Gender**			0.404
Female	10 (19.2%)	5 (50.0%)	
Male	42 (80.8%)	15 (35.7%)	
**Age (years)**			0.264
<60	31 (59.6%)	10 (32.3%)	
≥60	21 (40.4%)	10 (47.6%)	
**Tumor type**			0.079
Primary tumor (HCC and CRC)	36 (69.2%)	11 (30.6%)	
MLC	16 (30.8%)	9 (56.3%)	
**Smoking history**			0.350
No	35 (67.3%)	15 (42.9%)	
Yes	17 (32.7%)	5 (29.4%)	
**History of alcohol consumption**			1.000
No	39 (75.0%)	15 (38.5%)	
Yes	13 (25.0%)	5 (38.5%)	
**Tumor size (cm)**			0.143
<5.2	30 (57.7%)	9 (30.0%)	
≥5.2	22 (42.3%)	11 (50.0%)	
**Blood type**			0.008**
O	13 (25.0%)	9 (69.2%)	
Non-O (A, B, and AB)	39 (76.0%)	11 (28.2%)	
**Vascular invasion**			0.924
No	36 (69.2%)	14 (38.9%)	
Yes	16 (30.8%)	6 (37.5%)	
**Perineuronal invasion**			1.000
No	30 (57.7%)	10 (33.3%)	
Yes	3 (5.8%)	1 (33.3%)	
NA	19 (11.5%)	9 (47.4%)	
**Differentiation**			0.583
Moderate	32 (61.6%)	11 (34.4%)	
Poor	14 (26.9%)	6 (42.9%)	
NA	6 (11.5%)	3 (50.0%)	
**TNM stage**			0.008**
I-II	25 (48.1%)	5 (20.0%)	
III-IV	27 (51.9%)	15 (55.6%)	
**Lymph node metastasis**			0.120
Present	17 (32.7%)	9 (52.9%)	
Absent	30 (57.7%)	9 (30.0%)	
NA	5 (9.6%)	2 (40.0%)	
**Distant metastasis**			0.102
Present	17 (32.7%)	9 (52.9%)	
Absent	31 (59.6%)	9 (29.0%)	
NA	4 (7.7%)	2 (50.0%)	
**CEA (0-5 ng/ml)**			0.015*
Normal	34 (65.4%)	9 (26.5%)	
Abnormal	18 (34.6%)	11 (61.1%)	
**CA19-9 (0-37 U/ml)**			0.017*
Normal	29 (55.8%)	7 (24.1%)	
Abnormal	23 (44.2%)	13 (56.5%)	
**CA72.4 (0-6.7 U/ml)**			0.264
Normal	31 (59.6%)	13 (41.9%)	
Abnormal	21 (40.4%)	7 (33.3%)	
**CA242 (0-20 U/ml)**			0.135
Normal	35 (67.3%)	11 (31.4%)	
Abnormal	17 (32.7%)	9 (52.9%)	

CRC, colorectal carcinoma; HCC, hepatocellular carcinoma; MLC, metastatic liver cancer; NA, not available; *p<0.05, **p<0.01. Chi-square test.
