# Supplementary material for: Comprehensive comparison of patient-derived xenograft models in Hepatocellular Carcinoma and metastatic Liver Cancer
Source: Int J Med Sci. 2020 Oct 22;17(18):3073–81. doi: 10.7150/ijms.46686 (PMC7646096; doi:10.7150/ijms.46686)
Supplement: Supplementary file 1 — Supplementary tables. [file ijmsv17p3073s1.pdf]

Supplementary Table 1. Correlations between transplantation rate and clinicopathological parameters of HCC patients

Clinicopathological features	No. of patients (%)	Successful engraftment (%)	*P*
Gender			0.314
Female	6 (18.7%)	3 (50.0%)	
Male	26 (81.2%)	6 (23.08%)	
Age (years)			0.213
<60	21 (65.6%)	4 (19.1%)	
≥60	11 (34.4%)	5 (45.5%)	
Smoking history			0.704
No	19 (59.4%)	6 (31.6%)	
Yes	13 (40.6%)	3 (23.1%)	
History of alcohol consumption			0.685
No	23 (71.9%)	6 (26.1%)	
Yes	9 (28.1%)	3 (33.3%)	
History of hepatitis virus infection			1.000
No	4 (12.5%)	1 (25.0%)	
Yes	28 (87.5%)	8 (28.57%)	
Tumor size (cm)			1.000
<5.2	17 (53.1%)	5 (29.4%)	
≥5.2	15 (46.9%)	4 (26.7%)	
Blood type			0.001**
O	8 (25.0%)	6 (75.0%)	
Non-O (A, B, and AB)	24 (75.0%)	3 (12.5%)	
Vascular invasion			0.213
No	21 (65.6%)	4 (19.1%)	
Yes	11 (34.4%)	5 (45.5%)	
Perineuronal invasion			1.000
No	26 (81.3%)	8 (30.8%)	
Yes	2 (6.2%)	1 (50.0%)	
NA	4 (12.5%)	0 (0.0%)	
TNM stage			0.023*
I-II	24 (75.0%)	4 (16.7%)	
III-IV	8 (25.0%)	5 (62.5%)	
Differentiation			0.175
Moderate	18 (56.3%)	3 (16.7%)	
Poor	9 (28.1%)	4 (44.4%)	
NA	5 (15.6%)	2 (40.0%)	
Lymph node metastasis			0.042*
Present	4 (12.5%)	3 (75.0%)	
Absent	23 (71.9%)	4 (17.4%)	
NA	5 (15.6%)	2 (40.0%)	
AFP (0-7 ng/ml)			0.545

Normal	13 (40.6%)	4 (30.8%)	
Abnormal	19 (59.4%)	5 (26.3%)	
CEA (0-5 ng/ml)			0.076
Normal	25 (78.1%)	5 (25.0 %)	
Abnormal	7 (21.9%)	4 (57.1%)	
CA19-9 (0-37 U/ml)			0.049*
Normal	17 (53.1%)	2 (11.8%)	
Abnormal	15 (46.9%)	7 (46.7%)	
CA72.4 (0-6.7 U/ml)			0.681
Normal	21 (65.6%)	5 (23.8%)	
Abnormal	11 (34.4%)	4 (36.4%)	
CA242 (0-20 U/ml)			0.226
Normal	23 (71.9%)	5 (21.7%)	
Abnormal	9 (28.1%)	4 (44.4%)	

Fisher's exact test

Supplementary Table 2. Correlations between transplantation rate and clinicopathological parameters of MLC patients

Clinicopathological features	No. of patients (%)	Transplantation rate (%)	*P*
Gender			1.000
Female	2 (12.5%)	1 (50.0%)	
Male	14 (87.5%)	8 (57.1%)	
Age (years)			0.315
<60	8 (50.0%)	6 (75.0%)	
≥60	8 (50.0%)	3 (37.5%)	
Primary tumor type			0.809
Rectal cancer	6 (37.5%)	4 (66.7%)	
Colon cancer	8 (50.0%)	4 (50.0%)	
Others	2 (12.5%)	1 (50.0%)	
Smoking history			1.000
No	12 (75.0%)	7 (58.3%)	
Yes	4 (25.0%)	2 (50.0%)	
History of alcohol consumption			1.000
No	12 (75.0%)	7 (58.3%)	
Yes	4 (25.0%)	2 (50.0%)	
Tumor size (cm)			0.034*
<5.2	11 (68.8%)	4 (36.4%)	
≥5.2	5 (31.2%)	5 (100.0%)	
Blood type			0.585
O	4 (25.0%)	3 (75.0%)	
Non-O (A, B, and AB)	12 (75.0%)	6 (50.0%)	
TNM stage			-
I-II	0 (0.0%)	0 (0.0%)	

III-IV	16 (100.0%)	9 (100.0%)	
Lymph node metastasis			0.633
Present	10 (62.5%)	5 (50.0%)	
Absent	6 (37.5%)	4 (66.7%)	
CEA (0-5 ng/ml)			1.000
Normal	8 (50.0%)	4 (50.0%)	
Abnormal	8 (50.0%)	5 (62.5%)	
CA19-9 (0-37 U/ml)			0.358
Normal	9 (56.2%)	4 (44.4%)	
Abnormal	7 (43.8%)	5 (71.4%)	
CA72.4 (0-6.7 U/ml)			1.000
Normal	8 (50.0%)	4 (50.0%)	
Abnormal	8 (50.0%)	5 (62.5%)	
CA242 (0-20 U/ml)			0.633
Normal	10 (62.5%)	5 (50.0%)	
Abnormal	6 (37.5%)	4 (66.7%)	

Fisher's exact test

Supplementary Table 3. Correlations between transplantation rate and HBV-related antigen/antibody of HCC patients

Clinicopathological features	No. of patients (%)	Transplantation rate (%)	*P*
HBSAg			0.761
+	20	6	
-	12	3	
HBSAb			0.642
+	9	2	
-	23	7	
HBeAg			0.084
+	5	3	
-	27	6	
HBeAb			0.612
+	20	5	
-	12	4	
HBcAb			0.660
+	27	8	
-	5	1	
